# The technique of transforming symptom's symbol into emptiness: A mind–body therapy in the Chinese context

**DOI:** 10.1002/pchj.809

**Published:** 2024-11-07

**Authors:** Haiqun Niu, Yi Chen, Wen Zhou, Yanqiang Tao, Tianjun Liu

**Affiliations:** ^1^ School of Psychology Nanjing Normal University Nanjing China; ^2^ Yikong Skill Research Institute Nanjing China; ^3^ Faculty of Psychology Beijing Normal University Beijing China; ^4^ School of Acupuncture‐Moxibustion and Tuina Beijing University of Chinese Medicine Beijing China

**Keywords:** psychological nothingness, Qigong, TSSE

## Abstract

The technique of transforming symptom's symbol into emptiness (TSSE) is a new mind–body treatment method proposed by Tianjun Liu in 2008. It integrates Qigong and concrete object‐image thinking rooted in traditional Chinese culture into modern psychotherapy and proposes that mental and physical problems can be alleviated or eliminated in the process of movement. Accordingly, the therapist needs to guide the client with various symptoms to psychological nothingness where the client cannot see or feel these symptoms, and the purpose of healing can be achieved through the experience of emptiness. TSSE is divided into static and dynamic operations and consists of 10 steps. The static operation includes trio relaxation exercises (the body, breath, and mind), identifying the target symptom, visualizing the target symptom as an object‐image, visualizing a symbolic carrier, and filling out record sheet A. The dynamic operation includes trio relaxation exercises again, moving the symbolic object into the carrier, moving the carrier with the symbolic object into psychological nothingness, moving back and assessment, and filling out record sheet B. The effectiveness of TSSE can be evaluated by the therapist's judgment based on the client's performance and by the difference between the symptom impact scores recorded in sheets A and B. TSSE has been proven to be an effective psychosomatic treatment solution by some empirical studies conducted in China. Future research can combine other technologies, such as fMRI and fNIRS, to further explore the potential effective mechanisms of TSSE.

## INTRODUCTION

Mental health problems are now a higher global health priority than it was two decades ago (Patel, 2019). However, increasing social and economic inequalities, prolonged conflicts, and public health emergencies continue to impede progress in improving people's mental health (World Health Organization, 2022). This has also prompted researchers from various countries and fields to seek ways to maximize human welfare within their respective specialties. In the field of psychology, the continuous development of effective psychotherapeutic techniques has become a significant part of psychologists' work. However, as Western‐rooted psychotherapy techniques are introduced and practiced more widely in China, a growing number of Chinese scholars are recognizing the need to develop culturally appropriate approaches to enhance treatment effectiveness (e.g., Xiang et al., 2006; Yin et al., 2009; Zhang, 2019).

In this context, the technique known as transforming symptom's symbol into emptiness (TSSE), also referred to as the *yikong* skill (the Chinese word *yi* means “move,” and *kong* means “emptiness”), has emerged as a new mind–body approach grounded in traditional Chinese philosophy and culture. TSSE was initially presented by Tianjun Liu from the Beijing University of Chinese Medicine at the 5th World Congress for Psychotherapy in 2008, where it was introduced as the “moving‐box technique.” TSSE requires the therapist to guide the client in fully utilizing their imaginative capacity to symbolically concrete the target symptom that needs resolution, placing it into an imaginary carrier. Subsequently, the client is encouraged to repeatedly move the carrier at varying imagined spatial distances until entering psychological nothingness. In modern psychological terms, psychological nothingness can be understood as a state of conscious existence devoid of conscious content—a vast imagined space free from symptoms. TSSE emphasizes that symptoms can naturally diminish within psychological nothingness, and in the process of transforming, both negative emotional distress and physical sensations can be effectively alleviated or even eliminated (Liu, 2008). The current study aims to provide a detailed introduction to the origins and specific operational steps of TSSE.

## THE THEORETICAL BACKGROUND

### Qigong

Qigong, an integral part of traditional Chinese culture and heritage, refers to a practice that integrates body, breath, and mind adjustments into a harmonious and natural whole (Liu, 2010). *Qi* can be envisioned as an energy that flows throughout the body. *Gong*, meaning “practice,” resonates with its use in phrases like *gongli* (physical strength) and *gongfu* (or kungfu, meaning “power and capability”) (Ng, 1999). Therefore, Qigong is described as a method of “building vitality and warding off evils” (Jin et al., 2010). At its highest level, Qigong involves the integration of body, breath, and mind. All other distracting thoughts can be erased when this holistic integration is achieved.

Certain specific Qigong practices have been adopted by Western psychotherapy, such as Chanxiu (Hayes, 2002; Lane et al., 2007) and meditation techniques (Bowen et al., 2006; Foley et al., 2010) used in mindfulness training. Tianjun Liu, the founder of TSSE, emphasizes the significance of Qigong's mental adjustments and incorporates trio relaxation training from Qigong into TSSE. Therefore, the first step in TSSE involves three relaxation exercises (body, breath, and mind) to help clients reach a state of relaxation and peace. It is important to note that while Qigong emphasizes the integration of the three adjustments into a unified whole, TSSE focuses on coordinating these three elements without the same level of integration.

### Concrete object‐image thinking

The concept of concrete object‐image thinking (CO‐thinking) was first proposed in 1994 by Tianjun Liu (1994), who defined it as a purposeful cognitive activity wherein individuals process object‐images. In his perspective, the object‐image refers to the intuitive perceptual image of things, that is, the perception itself (Evans, 1991). Thus, CO‐thinking can be viewed as a high‐level thinking mode that is different from familiar abstract thinking and imagery thinking in which CO‐thinking directly processes individuals' perceptions, while the latter two utilize concepts and representations (i.e., the recollection of the intuitive perception of things) as their respective cognitive mediums. An example worth mentioning is that in Liu's view, the feelings in dreams are so realistic because the scenes in dreams are actually object‐images composed of real perceptions, rather than representations. To be more specific, taking the scene of “My left foot is soaked in warm water” as another example, reading this sentence (word concept) is abstract thinking, imagining this scene (representation) is imagery thinking, and experiencing the feeling of warmth in the left foot (object‐image) is CO‐thinking (Zhang & Liu, 2011).

CO‐thinking operations involve constructing and manipulating object images, with construction entailing forming an image of an imaginary object in consciousness and manipulation involving spatial–temporal and attribute operations on object images (Wei, 2009). For example, suppose a client, guided by a therapist, imagines intense inner conflict as an iron hook embedded in the body. In that case, the iron hook serves as an object‐image constructed through concrete thinking by the client. Subsequently, when the client purposefully changes the iron hook's location (a spatial–temporal operation) or attributes (a replacement operation), they manipulate the object‐image. However, Zhou and Liu (2020) also emphasize that throughout the practical application of TSSE, abstract thinking and imagery thinking are still involved, albeit with a primary emphasis on concrete object‐image thinking.

### Cognitive behavioral therapy

Cognitive behavior therapy (CBT) is widely regarded as the most thoroughly studied type of psychological treatment and is recommended in most treatment guidelines (Cuijpers et al., 2023). CBT has demonstrated effectiveness in treating various mental health problems, including depression (Cuijpers et al., 2013), insomnia disorder (Baglioni et al., 2023), and mood symptoms (Weintraub et al., 2023). As a mature psychotherapy approach with standardized treatment protocols, CBT serves as a model for the standardization of TSSE. TSSE, like CBT, is structured as a step‐by‐step therapy process. It involves identifying the problem to be addressed at the outset, formulating and implementing a plan, and evaluating effectiveness upon completion. However, the key distinction lies in the treatment focus: CBT targets changes in current cognitive behaviors, whereas TSSE aims to enhance clients' quality of life by guiding them to a state of psychological nothingness, where symptoms dissipate. This approach fosters unity of body and mind, ultimately liberating clients from their problems.

## THE CORE FEATURE OF TSSE

The core and most distinctive feature of TSSE is the concept of psychological nothingness. TSSE aims to guide clients with various physical and mental symptoms toward a state of psychological nothingness, where the experience of emptiness can facilitate healing. In contrast to Western ideals that emphasize action and substance, Eastern philosophy places greater significance on emptiness and the nonmaterial, suggesting that emptiness or the act of emptying ourselves allows us to fully engage with and submit to life (Yang, 2017, p. 10). Traditional Chinese culture holds numerous expressions highlighting the importance of recognizing the value of emptiness or nothingness. For example, in the classic Taoist text, the *Tao Te Ching*, there are passages such as “*Wu* is nothingness, emptiness, non‐existence… Only when it has *wu*, does it have life” (Lao‐tzu, 2003, p. 24). This wisdom, advocating for natural flow and nonaction, is widely embraced in China. Inspired by these principles, Liu creatively introduced TSSE as a mind–body technique more suitable for Chinese individuals, advocating that psychological nothingness serves as both the therapeutic method and goal. In the context of TSSE, moving toward emptiness holds dual meanings: guiding individuals toward nothingness and enabling them to experience nothingness through movement. Essentially, TSSE involves symbolically concrete symptoms and shifting them into psychological nothingness, rendering them invisible and unfelt to the client. As the symptoms move further away, the client gradually loses awareness of them, experiencing relief and a sense of nothingness in the process. This progression ultimately leads to healing.

While other therapeutic techniques can be likened to removing a spoonful of salt from a glass of water to reduce its saltiness, TSSE operates by dispersing that spoonful of salt into a vast lake (i.e., psychological nothingness) to achieve the same outcome. A more vivid metaphor would be comparing the target symptom to an iceberg. Traditional treatment methods aim to chip away at the iceberg, while TSSE achieves healing by melting the iceberg, and the act of melting the iceberg represents the role of psychological nothingness in this technique. Consequently, clients undergoing TSSE are not required to activate defense mechanisms (such as denial or repression) to cope with their psychological disorders. Instead, they are encouraged to directly acknowledge and assimilate these symptoms. This approach fosters acceptance and absorption of the symptoms within the context of psychological nothingness.

## THE OPERATIONAL STEPS OF TSSE

In 2019, the TSSE operational manual was successfully published and became an important guide for training therapists who want to use this technique (Liu, 2019). According to this, TSSE can be divided into static and dynamic operations, and each operation consists of five specific steps.

### The static operation

- Trio relaxation exercises

Some relaxation exercises for the body, breath, and mind are essential prerequisites for TSSE. For body relaxation, the client is instructed to sit upright in the front third of the chair, place both hands on the thighs, and lightly close their eyes. Regarding breath relaxation, the client focuses solely on exhaling instead of inhaling, guided by the therapist, and is encouraged to take three to five deep, slow, and smooth breaths. For mind relaxation, the client is directed to synchronize their exhaling rhythm with the expulsion of all awareness, thoughts, and emotions from the mind. This step typically lasts for 3–5 min, calming the client and allowing them to focus fully on the present moment, facilitating effective progression to subsequent operations.

ii Identify the target symptom

As clients often present with multiple issues, this step requires the therapist to assist them in identifying the core problem that needs prioritized resolution through a series of guiding questions. As mentioned earlier, TSSE can address both negative psychological emotions (e.g., anxiety, fear) and negative physical sensations (e.g., headache, chest tightness). Therefore, the client will be asked to specify which type of emotion or physical sensation, as well as which part of the body, troubles them the most. Once the specific target symptom has been well identified, the next substep involves rating its impact on a scale from 0 to 10. It is important to note that the impact of a symptom refers not only to its severity but also to the extent of interference with the client's mind and body. Although there may not always be a strict distinction between these aspects, optimal efficacy is typically achieved in clinical practice by addressing one symptom rated 7 or higher (or at least 5) at a time. Furthermore, if multiple symptoms are present, it is possible to address two to three similar symptoms concurrently as long as their combined score exceeds 7, even if no individual symptom scores are higher than 5.

iii Visualize the target symptom as an object‐image

Once the target symptom is selected, the client is guided to visualize it as a concrete object‐image with distinct physical properties. Initially, the therapist uses inductive questioning to help the client naturally form a symbolic representation of this target symptom. These symbolic objects can theoretically be anything, but in clinical practice, they often relate to common items from the client's daily life and living environment. For example, chest tightness might be visualized as a stone, or a person experiencing anger might imagine it as a fire. Unlike psychoanalysis, TSSE emphasizes the mobility of symptoms toward nothingness rather than delving into their origins or subconscious meanings. Therefore, the therapist ensures that these symbolic objects are vivid and realistic to facilitate their movement into a container (as detailed in the subsequent steps). There are two methods to achieve this transformation. The first one is detail‐inducing questioning. The client is prompted to describe specific details such as size, shape, weight, sound, and texture associated with the symbolic object, enhancing the clarity of its image (Tao et al., 2022). The second one is sensory‐inducing questioning: The therapist asks sensory‐related questions involving visualization, auditory perception, olfactory (smell), and tactile (touch) sensations to further enhance the realism of the symbolic object.

iv Visualize a symbolic carrier

TSSE treats symbols as expressive representations of symptoms, where the carrier symbolizes the inner strength of the client to address these symptoms. In this step, the client is tasked with using their imagination to create a carrier. Any object that holds or contains symbolic items can serve as a carrier, such as a box, cup, envelope, or even a shovel. However, it is important to ensure that the chosen carrier aligns with the symbolic object. If there is a mismatch—for instance, placing a stone in a paper bag or a flame in a wooden box—the therapist should guide the client to consider repairing or replacing the carrier to prevent issues during movement. For example, the therapist might suggest “Wouldn't it be better to use a wooden box for the stones?” Next, the client is encouraged to vividly imagine the carrier through detailed and sensory‐related questions, enhancing the clarity and realism of the carrier‐image. This process helps solidify the mental representation of the carrier, optimizing its effectiveness in the subsequent stages of TSSE.

v Fill out record sheet A

The final step of the static operation is to document the symbolic object and carrier on record sheet A, enhancing the client's memory of these object‐images and providing a foundation for evaluating therapeutic efficacy later on. The therapist can encourage the client to include as many details as possible in their drawings, specifying three to five key features (such as color, shape, weight, etc.) that they consider most important. This entire process typically takes about 5 min to complete.

### The dynamic operation

- Trio relaxation exercises again

Before initiating movement, the client is guided through a brief round of relaxation exercises similar to those described earlier.

ii Move the symbolic object into the carrier

The client is instructed to carefully inspect and clean both the symbolic object and the carrier from all angles and in appropriate ways. If the carrier is a container, thorough cleaning inside and outside is advised. Next, the client imagines placing the symbolic object into the carrier, following the opening, placing, arranging, and closing sequence. Throughout this process, the therapist asks questions about the positioning of the symbolic object, the available space inside the carrier, and its mobility to ensure proper placement. The therapist also reminds the client to secure or reinforce the carrier for smooth movement ahead.

iii Move the carrier with the symbolic object into psychological nothingness

**FIGURE 1:**
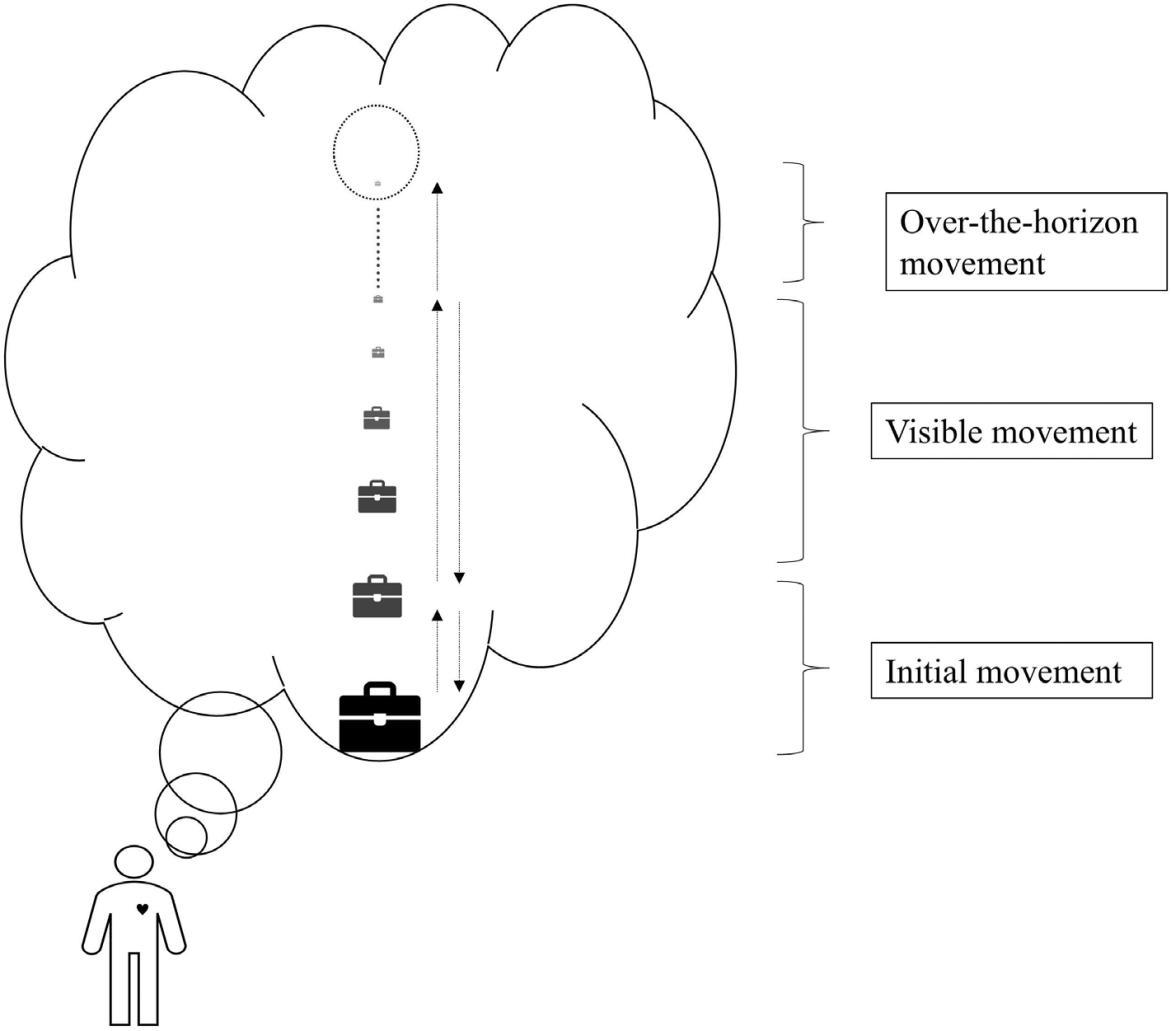
Moving symptom's symbol into psychological nothingness.

This step represents the core therapeutic process of TSSE (see Figure 1) and involves three substeps based on different movement distances: initial movement, visible movement, and over‐the‐horizon movement. Before initiating movement, the client needs to be informed to (1) keep all movements within their field of view at eye level, avoiding side‐to‐side or up‐and‐down movements, and (2) maintain focus solely on the Carrier (short for the carrier with symbolic object) within their field of view, excluding other visual distractions, and (3) have the option to keep eyes closed during the process, although opening and closing the eyes are permissible, and (4) establish an agreed‐upon signal (e.g., nodding or lifting a specific finger) to indicate movement to specified distances.

- Initial movement
  Initial movement entails short‐distance back‐and‐forth movement ranging from 1 to 3 m in front of the client's eyes. The therapist guides the client to position the Carrier within view and oscillate it between 1 and 3 m for two to three rounds. Throughout this phase, the therapist monitors each movement following the client's confirmation of clear visibility and inquires about the client's psychosomatic experiences during the process.
- Visible movement
  Visible movement involves longer back‐and‐forth movements at varying distances within the client's visible range. Following the initial movement, the therapist instructs the client to repeat these motions approximately 10 times at a further distance, identifying an optimal comfort distance based on client feedback. The therapist will suggest pausing for a moment at this optimal distance and ask the client how they are feeling. Then this phase continues with movements extending beyond 10 times to enhance the client's sense of control and determine the farthest distance at which the Carrier appears as a dot. Distances and corresponding sensations are recorded.
- Over‐the‐horizon movement
  The over‐the‐horizon movement extends beyond visible distances into psychological nothingness, where symptoms cease to exist. Movements may span units of 10 or 100 m, mainly progressing forward to minimize back‐and‐forth motions. Unlike previous movements focused on the physical act, this stage emphasizes the experience of emptiness (*kong* in Chinese). Psychological nothingness is more than just a location out of psychological view; it implies a status of experiencing a total absence of symptoms. Thus, the negative symptoms will be alleviated or disappear when the client gets into psychological nothingness. Accordingly, the therapist engages the client in dialogue about their feelings during movement toward psychological nothingness until the carrier is out of sight. The therapist may ask the client guiding questions as appropriate. For example, “even though you can no longer see the moving object, do you still feel it is there?” or “If you can't see it and feel that it is gone, is there anything lingering in your mind?” These questions help lead the client toward psychological nothingness. The client remains in psychological nothingness for 1–3 min.

iv Move back and assessment

This step may be omitted if the client indicates no need to retract the Carrier. Otherwise, the client gradually retraces movements back toward the front of the eye, transitioning from psychological nothingness to 1 km, 0.5 km, 100 m, 20 m, 10 m, 3 m, 5 m, 1 m, and finally to the starting position. Throughout this process, the client closely observes the symbolic object and carrier, providing detailed descriptions of any changes observed in size, weight, color, shape, etc.

v Fill out record sheet B

The final step of the dynamic operation involves completing record sheet B, rating the impact of the session on a scale of 0–10, and documenting detailed changes observed in the two object‐images.

## THE ASSESSMENT CRITERIA OF EFFECTIVENESS AND EMPIRICAL SUPPORT

### The assessment criteria for effectiveness

The effectiveness of TSSE can be evaluated through both qualitative and quantitative assessments. Qualitatively, the therapist judges whether the client's symptoms have improved based on changes observed in the object‐image and carrier: An empty carrier and disappeared object‐image indicate complete symptom elimination and clinical cure. If the carrier and object‐image shrink, it signifies a reduction in symptom impact on the client. Changes in the form of the carrier and object‐image may reflect corresponding changes in symptom impact.

Quantitatively, the assessment is based on symptom impact scores recorded by the client on record sheets A and B. A rating of 0 on sheet B indicates a clinical cure. TSSE is considered highly effective if the impact level decreases by 1/2 or more (e.g., from level 8 on sheet A to level 4 and below on sheet B). TSSE is effective if the impact level decreases by 1/3 or more (e.g., from level 6 to level 4). TSSE is deemed ineffective if the impact level decreases by less than 1/3 (e.g., from level 5 to level 4). Regardless of qualitative or quantitative assessment, clients are expected to honestly and seriously report their true changes following TSSE treatment.

### The empirical support

Some proponents who align with Liu, the originator of TSSE, have applied TSSE in clinical practice over the past few years and have observed significant therapeutic effects. For example, Xia et al. (2016) conducted a 6‐week controlled trial using a stress reduction group counseling program based on TSSE. They divided the 24 recruited students into an experimental group and a control group (with an equal number of males and females in each group) through interviews and scale assessments. The experimental group received 2 hours of TSSE counseling per week for a duration of 6 weeks, while the control group did not receive any intervention. The results showed that college students participating in the program once a week had notably lower scores on self‐assessed stress perceptions and symptoms, along with higher self‐efficacy scores, compared with the control group, where no significant changes were observed. Importantly, follow‐up evaluations revealed that the intervention's effects persisted even 1 month after the program concluded. Similarly, Yeung et al. (2021) utilized TSSE to treat a patient with a traumatic experience, achieving remarkable results with distress scores decreasing from level 8 to level 2. Most notably, the patient reported improved emotional regulation and communication with their parents post‐treatment. Another notable study by Tao et al. (2022) explored TSSE's application in 107 patients treated by 17 therapists. All the recruited participants experienced mental distress, including headaches, fear and sadness, during the COVID‐19 pandemic, and they were asked to rate the influence of their symptoms before and after therapists used TSSE to intervene. By comparing the difference between the two, Tao et al. found that TSSE effectively alleviated clients' target symptoms and could be considered a psychotherapeutic intervention for individuals experiencing bereavement.

In addition to support from published research results, Liu's team has established a case database for TSSE and is conducting studies with larger sample sizes. More research results will take time to emerge. In addition, the “Mind Flower Project,” led by Dr. Chaogan Yan of Chinese Academy of Sciences, incorporates the application of TSSE in its approach to combating depression. Recent research progress indicates promising outcomes. A preliminary study involving 15 depressed patients revealed significant reductions in overall anxiety and depression levels following an 8‐week psychological intervention utilizing TSSE. Notably, participants exhibited decreased functional connectivity in brain networks, resembling effects seen with conventional drug treatments for depression (L. Li et al., 2021). By integrating TSSE with cutting‐edge brain imaging technology, the project has laid a scientific foundation for the efficacy of this innovative mind–body therapy. These initial findings bolster the prospect of extending TSSE's benefits to a broader demographic. Moreover, TSSE was also promoted in Germany by the past president of the International Hypnosis Society, Bernhard Trenkle, who facilitated the publication of the German version of the operational manual in 2021. In his correspondence with Liu, he mentioned that he had been using TSSE in his treatment and consultation practice for the past decade, and successfully used this technique to help clients with sleep disorders and chronic pain (Liu, 2019, pp. 151–156). He also creatively combined TSSE with eye movements to treat clients, achieving significant therapeutic results (Liu, 2019, pp. 163–172).

## CONCLUSION

This article offers a comprehensive introduction to the technique of transforming symptom's symbol into emptiness, covering background, characteristics, specific steps, assessment criteria, and more. TSSE represents an original mind–body treatment approach that integrates key concepts from traditional Chinese culture into modern psychotherapy. Its standout feature is the creation of a psychologically transformative space with potent healing effects. Since its inception over a decade ago, TSSE's efficacy has been validated through extensive practical applications and supported by advancements in modern science and technology. Leveraging China's rich traditional culture, TSSE holds promise as a straightforward, efficient, and effective psychosomatic treatment solution for enhancing human physical and mental well‐being. Moving forward, research results with larger samples and more diverse cultural backgrounds are also needed to further clarify its cross‐cultural validity and effectiveness. Researchers can also explore enhanced integration of TSSE with other psychotherapeutic techniques to optimize disorder treatment for clients. Additionally, further elucidating the physiological and psychological mechanisms underlying TSSE's effects will deepen our understanding of its therapeutic impact.

## CONFLICT OF INTEREST STATEMENT

The authors declare no conflicts of interest.

## ETHICS STATEMENT

This article does not contain any studies with human participants or animals performed by the author.

## Supporting information


**Data S1.** Supporting information.
