# Supplementary material for: The technique of transforming symptom's symbol into emptiness: A mind–body therapy in the Chinese context
Source: Psych J. 2024 Nov 7;14(2):172–8. doi: 10.1002/pchj.809 (PMC11961237; doi:10.1002/pchj.809)
Supplement: Supplementary file 1 — Data S1. Supporting information. [file PCHJ-14-172-s001.docx]

**Record Sheet A**

1. Please rate how much the symptom affects you.

moderate impact

greatest impact

no impact

|  |  |  |  |  |  |  |  |  |  |
| --- | --- | --- | --- | --- | --- | --- | --- | --- | --- |

10

9

8

7

6

5

4

3

2

1

0

1. Please visualize your symptom into a specific image and draw it.

Basic information of this image:

Name: ______ Quantity: ______ Color: ______ Size: ______ Others: ______

1. Please visualize a symbolic carrier and draw it.

Main features of this carrier (be as detailed as possible and fill in at least three items):

Size: Length ____ width ____ height ____ cm Weight: ____kg

Shape: ____ Material: ____ Hardness: ____ Texture: ____

Gloss: ____ Smell: ____ Style: ____ Decoration: ____

Others: ________

**Record Sheet B**

1. Please rate how much the symptom affects you.

moderate impact

greatest impact

no impact

|  |  |  |  |  |  |  |  |  |  |
| --- | --- | --- | --- | --- | --- | --- | --- | --- | --- |

10

9

8

7

6

5

4

3

2

1

0

1. Please briefly draw or write the changes in the object-image.

Basic information of the changes:

Name: ______ Quantity: ______ Color: ______ Size: ______ Others: ______

1. Please briefly draw or write the changes in the symbolic carrier.

Main features of this carrier (be as detailed as possible and fill in at least three items):

Size: Length ____ width ____ height ____ cm Weight: ____kg

Shape: ____ Material: ____ Hardness: ____ Texture: ____

Gloss: ____ Smell: ____ Style: ____ Decoration: ____

Others: ________

1. Personalized events during the treatment (filled in by the therapist).
